# A combined model based on clinical, radiomics, and deep transfer learning features for differentiating endometrial hyperplasia with polyps from endometrial cancer

**DOI:** 10.3389/fonc.2026.1818266

**Published:** 2026-07-28

**Authors:** Min Xu, Wei hua Xu, Zhao yang Lu, Chun ming Shen, Jing Yang

**Affiliations:** 1 Department of Ultrasound, Suzhou Ninth People’s Hospital, Suzhou, Jiangsu, China; 2 Department of Ultrasound, Jiangsu Shengze Hospital, Suzhou, Jiangsu, China; 3 Oncology Center, Jiangsu Shengze Hospital, Suzhou, Jiangsu, China

**Keywords:** deep learning radiomics, endometrial cancer, machine learning, predictive model, transvaginal ultrasound imaging

## Abstract

**Objective:**

This study aimed to develop an ultrasound-based deep learning radiomics nomogram for differentiating endometrial hyperplasia with polyps from endometrial cancer in endometrial lesions and to explore its clinical diagnostic efficacy.

**Methods:**

We retrospectively collected clinical data from patients who underwent transvaginal ultrasound examinations in Suzhou Ninth People’s Hospital and Jiangsu Shengze Hospital from January 2020 to October 2024. Various machine learning models were developed using clinical data, handcrafted radiomics features, and deep learning features, with the highest AUC model selected as optimal. Univariate and stepwise multivariate analyses identified significant clinical features, which were combined with deep learning radiomics to create a Combined Model. The model’s predictive performance was evaluated using ROC, calibration, and decision curves, along with a deep learning radiomics nomogram.

**Results:**

This retrospective study of 340 patients from two hospitals included 149 endometrial cancer cases. Among them, 305 patients were from Suzhou Ninth People’s Hospital and 35 were from Jiangsu Shengze Hospital. The 305 patients from Suzhou Ninth People’s Hospital were divided into a training cohort and an internal validation cohort in a 7:3 ratio, comprising 213 and 92 patients, respectively. The 35 patients from Shengze Hospital served as the external testing cohort. Multivariate analysis confirmed four independent predictors: Testosterone, Estradiol, Abnormal Bleeding, and Menopausal Status, used to build the final Clinical Model. The effectiveness of the models was assessed by comparing the area under the receiver operating characteristic curve (AUC) based on the internal validation cohort. The results showed that the Combined Model achieved an AUC of 0.991 (95% confidence interval [CI]: 0.980 - 1.000) on the training cohort, 0.94 (95% CI: 0.892 - 0.987) on the internal validation cohort, and 0.955 (95% CI: 0.882 - 1.000) on the external testing cohort. Calibration curves and DeLong test confirmed the Combined Model’s superior accuracy and performance over single-modality models, with DCA showing highest net benefit, and nomogram enabling individualized risk stratification.

**Conclusion:**

The ultrasound-based combined model has high clinical diagnostic value for diagnosing endometrial cancer and simple endometrial hyperplasia with polyps.

## 1 Introduction

Endometrial cancer (EC) is one of the most common malignant tumors in the female reproductive system. Its incidence continues to rise globally, and mortality rates are increasing annually (1, 2). The development of endometrial cancer is complex, involving genetic, environmental, and lifestyle factors, and is significantly influenced by metabolic syndrome, including obesity, diabetes, and hypertension (3, 4). Reproductive factors like hormone replacement therapy, early menarche, late menopause, and infertility are also considered associated with the occurrence of endometrial cancer (5, 6). Postmenopausal bleeding serves as the cardinal symptom of EC (7); however, it is non-specific, as it frequently mimics benign conditions such as uterine fibroids or endometriosis, necessitating accurate differential diagnosis (8). Notably, endometrial hyperplasia, particularly when presenting with polyps, shares overlapping clinical symptoms and imaging features with EC, making preoperative differentiation challenging yet crucial for determining the appropriate surgical approach and extent. Early identification and treatment of endometrial cancer can significantly improve patient survival rates, making attention to symptoms like abnormal vaginal bleeding crucial (1). Clinically, the diagnosis of EC typically relies on medical history, physical examination, and imaging studies. Imaging techniques such as ultrasound and MRI help evaluate endometrial thickness and tumor extension (9). Furthermore, pathological examination is the gold standard for diagnosis. Microscopic pathological examination of sampled tissues clarifies the tumor type and grade, guiding subsequent treatment (10).

Radiomics is an emerging field that analyzes tumors by extracting a large number of quantitative features from medical images. These radiomic features are often not directly observable by the human eye. The goal of radiomics is to represent these features as high-dimensional data for subsequent analysis and model building. The basic radiomics workflow includes image acquisition, segmentation of the region of interest (ROI), image preprocessing, feature extraction, feature analysis and selection, and finally, predictive model development (11). In tumor diagnosis, radiomics has achieved significant results. For instance, studies on various tumor types such as breast cancer, lung cancer, and prostate cancer have shown that radiomics models perform excellently in early tumor detection and grading (12–14). Specifically, regarding transvaginal ultrasound (TVUS), recent research indicates that deep learning algorithms can effectively capture texture and morphological nuances to distinguish between benign and malignant endometrial lesions, offering a more objective assessment compared to conventional visual inspection (15).

To overcome the limitations of previous studies, which often relied on isolated clinical parameters and provided limited insight into model decision-making, we developed a multimodal machine learning framework for differentiating endometrial hyperplasia with polyps from endometrial cancer. First, we systematically evaluated 18 clinical and laboratory variables, including demographic characteristics, tumor markers, and sex hormone profiles, to identify independent predictors of endometrial cancer. We then integrated these clinical features with ultrasound-based handcrafted radiomics and deep transfer learning features to construct a Combined Model. The optimal model architecture was selected according to the highest area under the receiver operating characteristic curve (AUC) in the internal validation cohort. In addition, Gradient-weighted Class Activation Mapping (Grad-CAM) was applied to visualize the image regions that contributed most to the model predictions. Finally, model discrimination, calibration, clinical utility, and interpretability were comprehensively assessed using receiver operating characteristic (ROC) curves, calibration curves, decision curve analysis, and a deep learning radiomics nomogram.

## 2 Materials and methods

### 2.1 Study cohort and clinical characteristics

#### 2.1.1 Patient cohort and data collection

**Figure 1:**
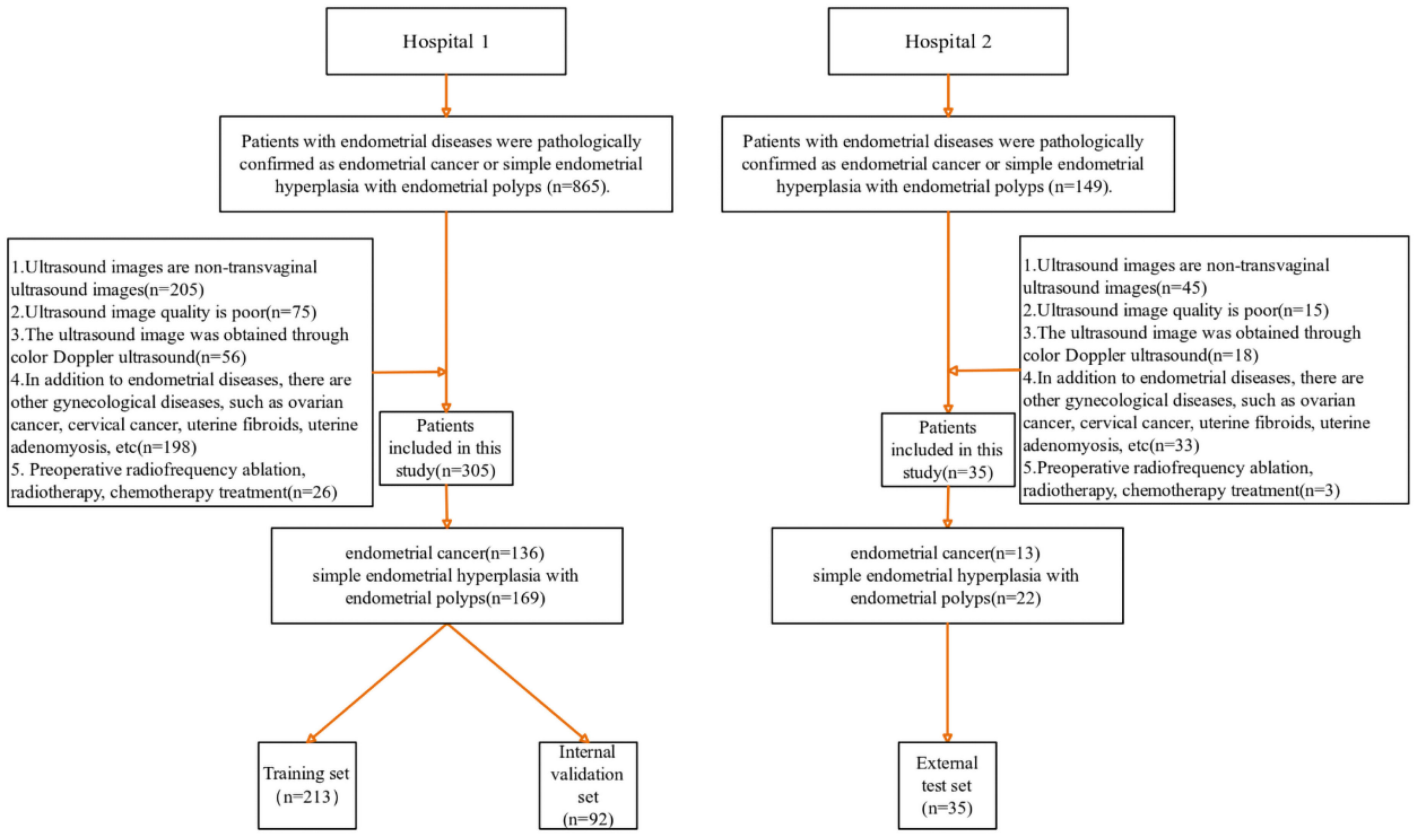
Flowchart of patient recruitment and study design.

This retrospective study analyzed data from patients who underwent transvaginal ultrasound examinations between January 2020 and October 2024, selected from Suzhou Ninth People’s Hospital and Jiangsu Shengze Hospital. The inclusion criteria were as follows: (1) First-time diagnosis; (2) Underwent transvaginal ultrasound examination; (3) Participants must have pathologically confirmed endometrial hyperplasia with polyps or endometrial carcinoma. (4) Complete and high-quality clinical, imaging, and pathological data. Exclusion criteria were: (1) Other gynecological diseases (e.g., ovarian cancer); (2) Poor-quality ultrasound images; (3) Preoperative radiotherapy, chemotherapy, or radiofrequency ablation; (4) No transvaginal ultrasound examination performed. All clinical variables were obtained through routine preoperative assessments before pathological diagnosis to ensure the model’s applicability in real clinical settings. The patient recruitment process is shown in **Figure 1**.

#### 2.1.2 Screening for clinical predictors

All included patients had pathologically confirmed diagnoses. This study also collected pre-treatment data on testosterone, progesterone, estradiol, carbohydrate antigen 199 (CA 199), follicle-stimulating hormone (FSH), abnormal bleeding, and menopause. Clinically related risk factors were identified using univariate and multivariate analyses. The study protocol received ethical approval from the Suzhou Ninth People’s Hospital and Jiangsu Shengze Hospital, which granted a waiver of the requirement for informed consent due to the retrospective and anonymized nature of this study. All data were analyzed in an anonymized form, adhering to the principles of the Declaration of Helsinki.

### 2.2 Ultrasound image acquisition and radiomics feature extraction

#### 2.2.1 Ultrasound image acquisition

All enrolled patients underwent preoperative transvaginal ultrasound examinations using ultrasound equipment including Toshiba Aplio 500 (Japan) and Philips RPIQ5 (Netherlands). Physicians with 5 to 10 years of experience in gynecological ultrasound evaluation performed the examinations using a vaginal probe. After the patient assumed the lithotomy position, multi-planar continuous scanning was performed to obtain standard sectional images of the endometrial region. In accordance with clinical quality control standards, approximately 7–10 representative transvaginal ultrasound (TVUS) images were captured and archived for each patient. These images included standard sectional views (mid-sagittal and transverse planes of the uterine body, vaginal/cervical scans) and specific focal planes displaying the lesion’s internal echo characteristics, vascularity via color Doppler, and its relationship with the myometrium.

#### 2.2.2 Region of interest segmentation

Two physicians, each with over 10 years of experience and blinded to the pathological results, reviewed the transvaginal ultrasound images of enrolled patients using the Picture Archiving and Communication System (PACS). From the series of images collected, they specifically selected the single 2D gray-scale ultrasound image that displayed the maximum cross-sectional area of the tumor for each patient to ensure the best representation of tumor heterogeneity. They then saved the selected images in BMP format and converted them to NII format. The open-source software ITK-SNAP (version 3.8, available at http://www.itksnap.org/) was used to manually delineate the region of interest (ROI) covering the target endometrial area. Radiologist A, who has 5 years of experience in gynecological ultrasound diagnosis, delineated ROIs for all patients. Then, Radiologist B, with 10 years of experience, randomly selected 50 patients for repeated delineation. Both radiologists were blinded to the patients’ histopathological results. Two months later, Radiologist A re-delineated the ROIs for all patients. The intraclass correlation coefficient (ICC) was calculated for each feature to assess the inter- and intra-observer agreement, and features with ICC values below 0.75 were excluded.

#### 2.2.3 Handcrafted feature extraction and feature selection

In this study, radiomics feature extraction was performed using the PyRadiomics open-source Python package (version 3.0, http://pyradiomics.readthedocs.io). To comprehensively quantify tumor heterogeneity, handcrafted features were systematically extracted from each tumor region of interest (ROI). These features were categorized into three base groups: (1) morphological features, characterizing the shape and size of the tumor; (2) first-order features, describing the distribution of voxel intensities; and (3) texture features, quantifying spatial inter-relationships and structural patterns. The texture group specifically comprised the Gray-Level Co-occurrence Matrix (GLCM), Gray-Level Run Length Matrix (GLRLM), Gray-Level Size Zone Matrix (GLSZM), Gray-Level Dependence Matrix (GLDM), and Neighboring Gray Tone Difference Matrix (NGTDM).

To capture multi-scale and higher-order imaging information, we applied various image filters to the original images prior to feature extraction. These included 8 Wavelet decompositions, 3 types of 3D Local Binary Pattern (LBP-3D), Gradient, Square, Square Root, Logarithm, and Exponential filters. According to the PyRadiomics pipeline, morphological features were computed exclusively from the original images, whereas the first-order and texture features were calculated from both the original images and all 16 derived image types.

#### 2.2.4 Radiomics feature selection

Prior to feature selection and model training, all radiomics features were normalized using Z-score normalization. To strictly prevent data leakage, the normalization parameters (mean and standard deviation) were calculated solely from the training cohort and then independently applied to the internal validation and external testing cohorts.

Following normalization, a rigorous multi-step feature selection process was performed on the training cohort:

1. Stability and redundancy filtering: First, features with low inter-observer stability (ICC< 0.75) were removed. Subsequently, Spearman’s rank correlation coefficient was used to analyze the pairwise correlations among features. To reduce high redundancy, when the correlation coefficient between two features exceeded 0.9, only one feature was retained.
2. Recursive feature elimination (RFE): To maximize the representative capability of the retained features, we applied an RFE method based on a greedy algorithm. This approach iteratively evaluated and deleted the most redundant or least informative features from the set to further reduce dimensionality.
3. LASSO regression: Finally, Least Absolute Shrinkage and Selection Operator (LASSO) logistic regression with 10-fold cross-validation was used to determine the optimal penalty parameter (λ). Features with non-zero coefficients at the optimal λ were selected to construct the final radiomics signature (Rad-score).

### 2.3 Deep transfer learning model and feature compression

This study used 17 deep neural networks pre-trained on ImageNet, including DenseNet, ResNet, classic CNNs, lightweight models, and a Transformer (ViT), which were implemented in PyTorch using official pre-trained weights. Preprocessing involved selecting the largest tumor cross-sections, applying min-max normalization, and converting the images to pseudo-RGB format. Inception_v3 and ViT inputs were resized to 299×299 pixels, while all other models received inputs resized to 224×224 pixels. Fine-tuning was performed using the SGD optimizer over 100 epochs, with a batch size of 32, a learning rate of 0.01, and a dropout rate of 0.5. Details are in **Supplementary Table 1**.

We removed the fully connected classification layers and extracted features from the global average pooling layer. Raw feature vectors had dimensionalities ranging from 1,024 to 4,096. To standardize the feature space and reduce redundancy, Z-score normalization and PCA were applied. To rigorously prevent data leakage, both the Z-score normalization and PCA transformations were fitted exclusively on the training cohort. The derived parameters (mean, standard deviation, and PCA transformation matrix) were subsequently applied to independently normalize and transform the validation and external testing cohorts. The derived parameters were then used to normalize the validation and external testing cohorts. PCA retained the top 128 principal components, thereby standardizing the feature dimensionality across all samples. The optimal deep learning architecture was selected based on AUC in the internal validation cohort, with the external testing cohort kept blinded. After identifying the backbone, the 128 extracted features were further screened via LASSO regression, conducted solely on the training cohort. Features exhibiting non-zero coefficients constituted the final DTL signature, which was subsequently evaluated across all cohorts.

### 2.4 Deep learning radiomics feature construction

Based on the screened handcrafted radiomics features and the compressed 128-dimensional DTL features, our goal was to construct a unified Deep Learning Radiomics (DLR) signature. We adopted a standardized construction pathway. After feature screening and dimensionality reduction via the LASSO algorithm, the retained features were input into various machine learning classifiers to build predictive models. Comprehensive details regarding the evaluated machine learning algorithms and their specific hyperparameter configurations are provided in **Supplementary Table 1**. The best-performing classifier in the internal validation cohort was selected to derive the final DLR signature.

### 2.5 Combined model construction and nomogram development

We used clinical data containing conventional ultrasound features as the reference basis. First, clinical features were screened through baseline statistical analysis, and those with statistical significance (p< 0.05) were selected. The clinical signature was then constructed using the same machine learning models employed for building the radiomics signature. Subsequently, by integrating the clinical signature and the Deep Learning Radiomics (DLR) signature via multivariable logistic regression, the Combined Model was constructed.

To provide a quantitative and intuitive tool for individualized clinical decision-making, a deep learning radiomics nomogram was developed based on the Combined Model. During the nomogram construction, the integrated predictors were assigned specific scores ranging from 0 to 100 by normalizing their logistic regression coefficients. The predictor with the largest absolute coefficient was assigned the full 100 points, while other variables were scaled proportionally. Finally, for each patient, a “Total Point” was calculated by summing the scores of all included features. This total score was then mapped to a sigmoid-transformed scale to estimate the specific probability (ranging from 0.01 to 0.99) of differentiating endometrial hyperplasia with polyps from endometrial carcinoma.

The diagnostic efficacy and clinical utility of this nomogram were comprehensively tested across the cohorts. Discrimination was evaluated using the Area Under the Curve (AUC) of the Receiver Operating Characteristic (ROC). To assess the nomogram’s calibration performance, calibration curves were plotted to examine the agreement between predicted probabilities and actual pathological outcomes, followed by the Hosmer-Lemeshow goodness-of-fit test. Furthermore, a Decision Curve Analysis (DCA) plot was drawn to evaluate and quantify the net clinical benefit of the predictive model.

### 2.6 Statistical analysis

We first performed univariate analysis, followed by multivariate logistic regression to identify independent predictors. These predictors were then applied to the validation and testing cohorts. Specifically, the normality of clinical features was assessed using the Shapiro-Wilk test. Continuous variables were analyzed with the t-test or Mann-Whitney U test, while categorical variables were analyzed using the chi-square test. P-values greater than 0.05 indicate no significant differences.

**Figure 2:**
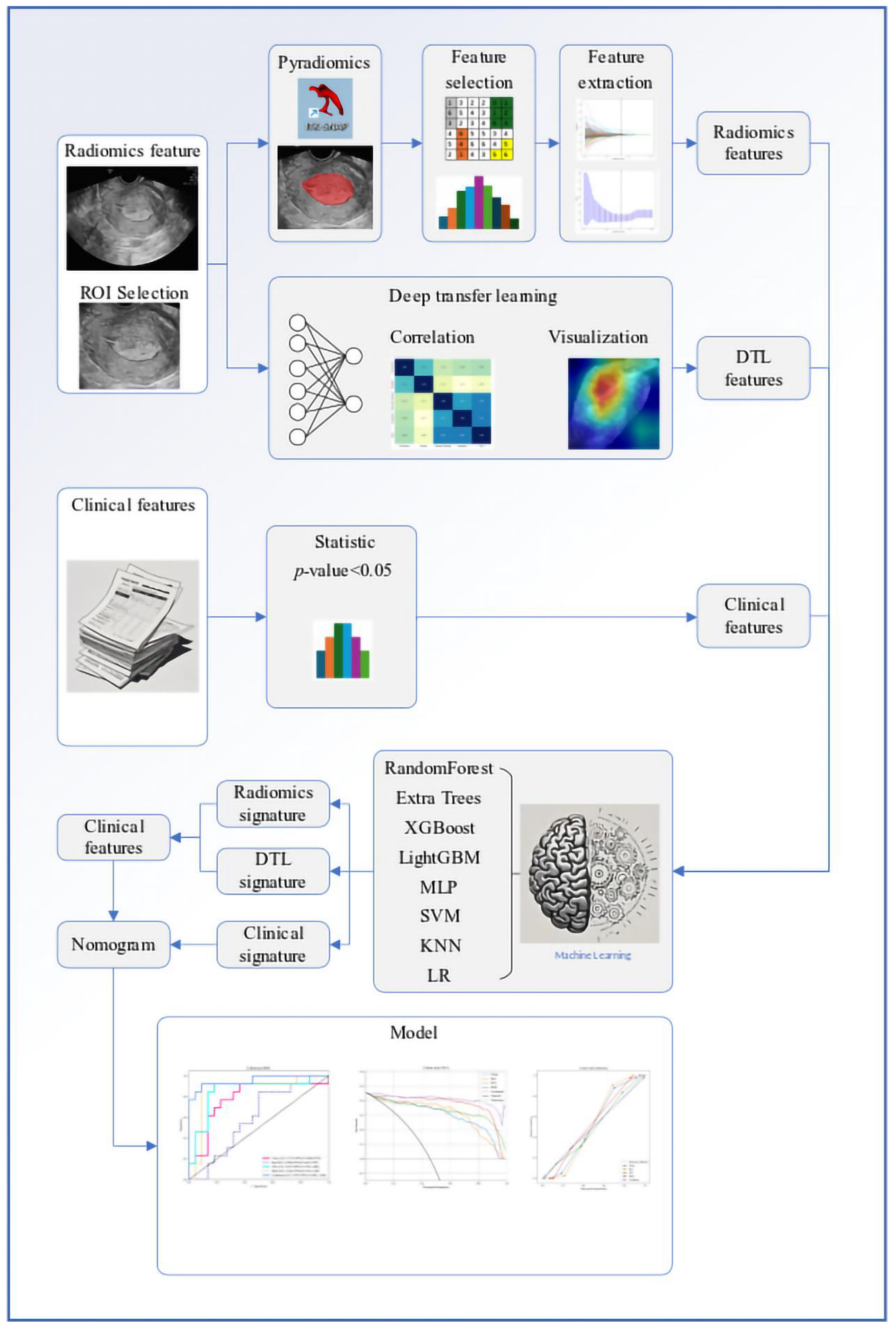
Schematic workflow of the model building process.

Data analyses were performed with Python 3.7.12 and Statsmodels 0.13.2 on the OnekeyAI 4.9.1 platform. Radiomics features were extracted using PyRadiomics 3.0.1. Machine learning models were built with Scikit-learn 1.0.2. Deep learning models were developed using PyTorch versions 1.8.1 to 1.11.0, optimized with CUDA 11.3.1 and cuDNN 8.2.1 on an NVIDIA 4090 GPU, and supplemented by MONAI 0.8.1. The process of model building is shown in **Figure 2**.

## 3 Results

### 3.1 Patient baseline characteristics

This retrospective study included 340 patients who underwent transvaginal ultrasound examinations at Suzhou Ninth People’s Hospital and Jiangsu Shengze Hospital between January 2020 and October 2024. Among them, 305 cases (89.7%) were from Suzhou Ninth People’s Hospital and 35 cases (10.3%) were from Shengze Hospital. Subsequently, the 305 cases from Suzhou Ninth People’s Hospital were divided into a training cohort and an internal validation cohort in a 7:3 ratio, comprising 213 and 92 cases, respectively. The 35 patients from Shengze Hospital served as the external testing cohort to evaluate the model’s generalization ability. Pathological confirmation identified 149 cases of endometrial cancer (136 from the Ninth Hospital and 13 from Shengze Hospital). The median age of the entire cohort was 48.9 (**Table 1**).

**Table 1:** Baseline clinical and pathological characteristics of patients in the training, validation, and testing cohorts.

Feature name	ALL	Training	Validation	Testing	P-value
Age	48.91 ± 12.28	48.54 ± 12.42	49.41 ± 12.72	49.77 ± 10.37	0.774
BMI	24.17 ± 3.74	24.41 ± 3.92	23.86 ± 3.30	23.54 ± 3.61	0.292
Age of menarche	14.01 ± 0.80	14.00 ± 0.85	14.09 ± 0.71	13.89 ± 0.76	0.418
Menopause	0.33 ± 0.47	0.32 ± 0.47	0.35 ± 0.48	0.34 ± 0.48	0.875
Abnormal bleeding	0.55 ± 0.50	0.54 ± 0.50	0.54 ± 0.50	0.60 ± 0.50	0.802
Gravida	1.54 ± 0.89	1.54 ± 0.89	1.54 ± 0.95	1.46 ± 0.74	0.861
Abortions	0.91 ± 1.07	0.84 ± 1.08	1.05 ± 1.01	0.91 ± 1.12	0.276
AFP	2.63 ± 2.06	2.74 ± 2.25	2.30 ± 1.60	2.85 ± 1.83	0.19
CEA	1.46 ± 1.01	1.46 ± 1.02	1.48 ± 1.03	1.48 ± 0.93	0.984
CA199	21.18 ± 87.50	24.03 ± 108.67	16.79 ± 28.28	15.39 ± 20.79	0.738
CA125	24.15 ± 39.95	23.55 ± 38.22	25.20 ± 43.66	25.07 ± 41.19	0.937
CA153	11.19 ± 22.49	11.66 ± 27.19	10.74 ± 12.27	9.56 ± 4.96	0.856
Testosterone	1.14 ± 0.69	1.13 ± 0.69	1.17 ± 0.69	1.10 ± 0.73	0.854
Prolactin	282.17 ± 281.11	273.27 ± 307.01	311.49 ± 257.47	259.29 ± 139.39	0.487
Progesterone	3.41 ± 7.85	3.59 ± 8.60	2.66 ± 5.04	4.27 ± 9.12	0.505
FSH	25.01 ± 27.92	24.08 ± 26.75	24.92 ± 29.27	30.94 ± 31.19	0.404
LH	44.87 ± 544.54	62.79 ± 687.90	14.19 ± 11.64	16.45 ± 15.21	0.735
Estradiol	301.80 ± 372.01	286.84 ± 357.53	316.45 ± 367.15	354.38 ± 466.01	0.554
FIGO stage, n (%)					
IA	95 (63.8%)	60 (63.2%)	28 (68.3%)	7 (53.8%)	
IB	29 (19.5%)	15 (15.8%)	10 (24.4%)	4 (30.8%)	
II	17 (11.4%)	13 (13.7%)	2 (4.9%)	2 (15.4%)	
III–IV	8 (5.4%)	7 (7.4%)	1 (2.4%)	0 (0.0%)	

BMI, body mass index; CA125, carbohydrate antigen 125; CA199, carbohydrate antigen 19-9; CEA, carcinoembryonic antigen; FSH, follicle-stimulating hormone; LH, luteinizing hormone; AFP, alpha-fetoprotein; CA153, carbohydrate antigen 15-3. FIGO, International Federation of Gynecology and Obstetrics.

### 3.2 Clinical feature correlation analysis

Based on the data from the training cohort, univariate analysis was performed on clinical variables to identify potential risk factors. In the univariate analysis, several clinical characteristics showed statistical significance (p< 0.05), including Testosterone, Progesterone, Estradiol, Carbohydrate Antigen 199 (CA199), Follicle-Stimulating Hormone (FSH), Abnormal Bleeding, and Menopausal Status.

Significant features identified through this preliminary screening were subsequently entered into a multivariate logistic regression analysis using the backward stepwise method. Collinearity diagnostics confirmed that the Variance Inflation Factor (VIF) for all selected variables was less than 5, indicating no severe multicollinearity. Further multivariate analysis identified four independent predictors that remained significant: Testosterone (OR = 0.452, 95% CI: 0.308-0.662, p=0.001), Estradiol (OR = 0.998, 95% CI: 0.997-0.999, p=0.015), Abnormal Bleeding (OR = 2.252, 95% CI: 1.276-3.971, p=0.019), and Menopausal Status (OR = 3.607, 95% CI: 1.744-7.456, p=0.004). These independent predictors were utilized to build the final Clinical Model (**Table 2**).

**Table 2:** Univariate and multivariate logistic regression analysis of clinical characteristics for predicting endometrial cancer in the training cohort.

Feature name	Univariate analysis	P value	Multivariate analysis	P value
	Odds ratios (95% CI)		Odds ratios (95% CI)	
Testosterone	0.806(0.676-0.961)	0.043	0.452(0.308-0.662)	0.001
Abortions	0.843(0.71-1.002)	0.104		
Progesterone	0.961(0.93-0.993)	0.049	0.996(0.963-1.03)	0.838
AFP	0.97(0.909-1.305)	0.433		
Gravida	0.98(0.863-1.112)	0.788		
Age of Menarche	0.989(0.973-1.005)	0.251		
BMI	0.994(0.985-1.004)	0.313		
Estradiol	0.998(0.998-0.999)	<0.001	0.998(0.997-0.999)	0.015
Prolactin	1.000(0.999-1.001)	0.928		
LH	1.000(0.998-1.001)	0.649		
Age	1.002(0.998-1.007)	0.43		
CA153	1.005(0.994-1.016)	0.433		
CA125	1.006(0.999-1.012)	0.16		
CA199	1.015(1.003-1.026)	0.034	1.017(0.996-1.038)	0.176
FSH	1.021(1.012-1.028)	<0.001	1.013(0.999-1.027)	0.129
CEA	1.143(1.002-1.303)	0.094		
Abnormal Bleeding	2.018(1.517-2.927)	<0.001	2.252(1.276-3.971)	0.019
Menopause	4.666(2.765-7.877)	<0.001	3.607(1.744-7.456)	0.004

OR, odds ratio; CI, confidence interval; Ref, reference category.

### 3.3 Radiomics feature selection and deep learning model construction

Through the comprehensive computational pipeline, a total of 1,561 handcrafted radiomics features were initially extracted from the tumor ROIs. This exact sum was yielded by calculating the combined 91 first-order and texture features across 17 image types (1 original + 16 derived), additionally supplemented by the 14 morphological features derived solely from original images (calculated as 91 features × 17 image types + 14 morphological features).

Following the multi-step feature selection process detailed in the Methods, the dimensionality of the feature space was progressively reduced (**Supplementary Figure 1**). The initial stability assessment, redundancy filtering, and the subsequent RFE process effectively narrowed down the initial 1,561 features to 143 robust candidates. Subsequently, applying the LASSO algorithm yielded an optimal penalty parameter (λ) corresponding to the minimum mean squared error (**Supplementary Figures 1A–D**). At this optimal λ, 10 key radiomics features with non-zero coefficients were finally selected to construct the Rad-score. The detailed list of these 10 features and their corresponding weights is provided in **Supplementary Table 2**.

For deep transfer learning (DTL), 17 candidate pre-trained architectures were systematically evaluated. ResNet152 was successfully identified as the optimal backbone due to its superior generalization and clinical applicability. It achieved the highest AUC of 0.908 (95% CI: 0.855–0.962) and a specificity of 0.960 (indicating a low false positive rate of 4.0%) in the internal validation cohort. Following feature extraction from ResNet152 and subsequent PCA dimensionality reduction, the refined DTL features were combined with the 10 handcrafted radiomics features.

To determine the optimal classification algorithm for the unified Deep Learning Radiomics (DLR) signature, multiple machine learning classifiers were compared (including LR, SVM, KNN, Random Forest, Decision Tree, XGBoost, LightGBM, and MLP). The comprehensive evaluation of these classifiers across the training and testing cohorts is detailed in **Table 3** and **Supplementary Table 3**.

**Table 3:** Performance comparison of different machine learning classifiers constructed using the deep learning radiomics (DLR) signature.

Model	Accuracy	AUC	95% CI	Sensitivity	Specificity	PPV	NPV	Cohort
LR	0.896	0.948	0.9184 -0.9767	0.9767	0.887	0.904	0.887	train
LR	0.85	0.903	0.8466 -0.9585	0.9585	0.846	0.853	0.8	test
SVM	0.929	0.976	0.9569 -0.9960	0.996	0.928	0.93	0.918	train
SVM	0.843	0.904	0.8475 -0.9607	0.9607	0.846	0.84	0.786	test
KNN	0.844	0.941	0.9130 -0.9697	0.9697	0.691	0.974	0.957	train
KNN	0.803	0.858	0.7893 -0.9276	0.9276	0.654	0.907	0.829	test
RandomForest	0.906	0.961	0.9382 -0.9839	0.9839	0.907	0.904	0.889	train
RandomForest	0.787	0.849	0.7760 -0.9217	0.9217	0.808	0.773	0.712	test
ExtraTrees	0.887	0.944	0.9148 -0.9732	0.9732	0.835	0.93	0.91	train
ExtraTrees	0.827	0.887	0.8242 -0.9501	0.9501	0.788	0.853	0.788	test
XGBoost	0.953	0.992	0.9841 -0.9990	0.999	0.938	0.965	0.958	train
XGBoost	0.866	0.91	0.8560 -0.9648	0.9648	0.769	0.933	0.889	test
LightGBM	0.91	0.969	0.9485 -0.9903	0.9903	0.897	0.922	0.906	train
LightGBM	0.874	0.908	0.8524 -0.9633	0.9633	0.904	0.853	0.81	test
MLP	0.915	0.962	0.9371 -0.9863	0.9863	0.876	0.948	0.934	train
MLP	0.835	0.875	0.8081 -0.9411	0.9411	0.75	0.893	0.83	test

LR, Logistic Regression, SVM, Support Vector Machine, RF, Random Forest, MLP, Multilayer Perceptron; AUC, area under the receiver operating characteristic curve; PPV, positive predictive value; NPV, negative predictive value; CI, confidence interval.

### 3.4 Grad-CAM visualization

**Figure 3:**
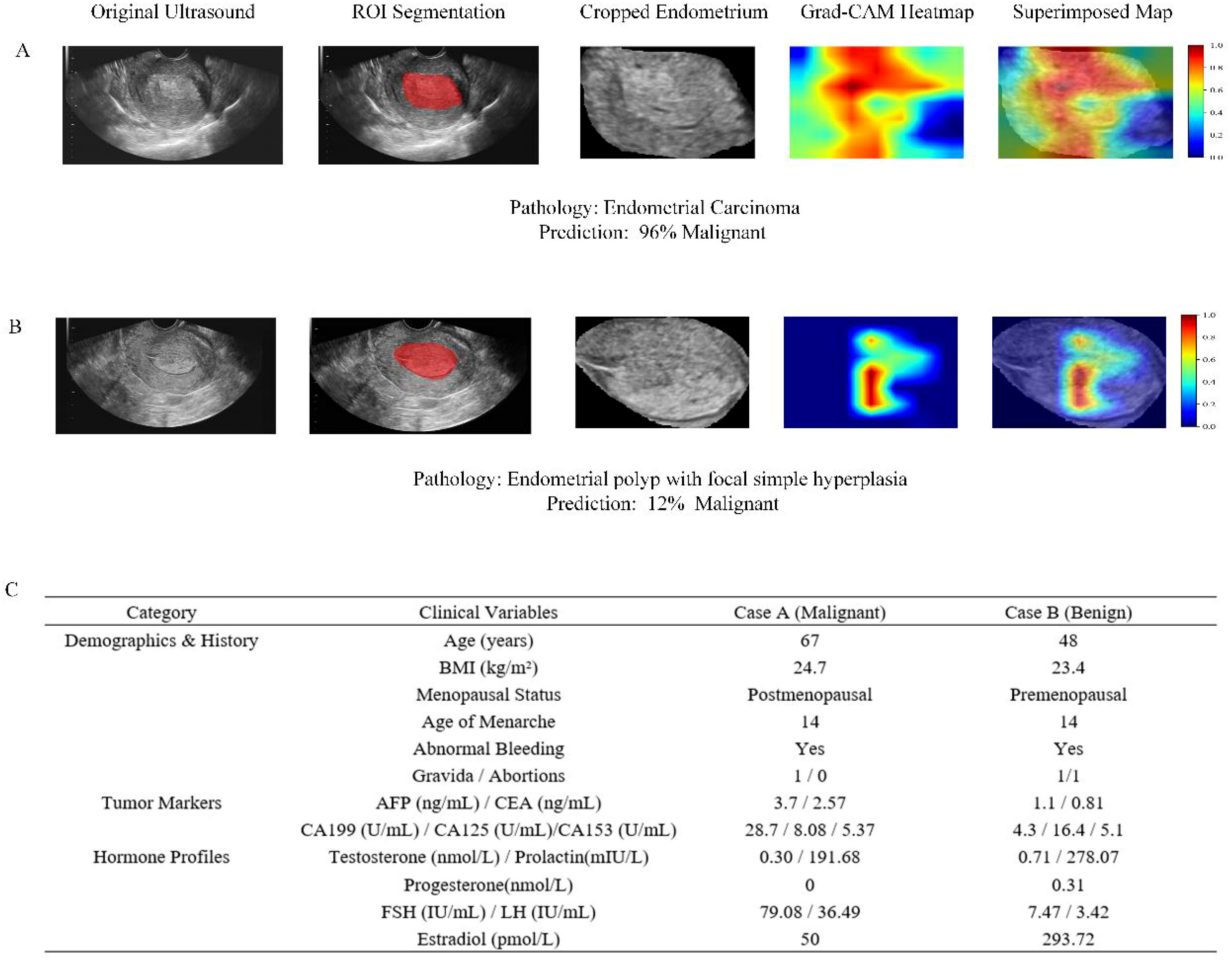
Representative Grad-CAM visualizations and comprehensive clinical characteristics of malignant and benign endometrial lesions. **(A)** Ultrasound images and Grad-CAM visualizations of an endometrial carcinoma (Malignant Case A). The model predicted a 96% probability of malignancy. From left to right: original ultrasound image, ROI segmentation (red mask), cropped endometrium, Grad-CAM heatmap highlighting the region’s most influential for the model’s decision, and the superimposed map. **(B)** Corresponding visualizations for an endometrial polyp with focal simple hyperplasia (Benign Case B), with a 12% predicted probability of malignancy. **(C)** Detailed comparison of clinical variables, including demographics, medical history, tumor markers, and hormone profiles for Case A and Case B ROI, region of interest; Grad-CAM, Gradient-weighted Class Activation Mapping; BMI, body mass index; AFP, alpha-fetoprotein; CEA, carcinoembryonic antigen; CA, cancer antigen; FSH, follicle-stimulating hormone; LH, luteinizing hormone.).

To further examine the prediction patterns of the best-performing deep learning model, Grad-CAM was applied to two representative cases. As shown in **Figure 3**, the malignant and benign lesions exhibited distinct attention patterns.

In the malignant case, which was pathologically confirmed as endometrial carcinoma, the model predicted malignancy with a probability of 96%. The highlighted regions were relatively diffuse and mainly distributed over areas with heterogeneous internal echotexture. In the benign case, diagnosed as an endometrial polyp with focal simple hyperplasia, the predicted probability of malignancy was 12%. In contrast, the highlighted regions were more focal and concentrated on localized structural abnormalities.

These visual findings suggest that the model primarily focused on lesion-related morphological and textural information when generating predictions, providing visual support for model interpretation.

### 3.5 Model effectiveness ROC curves

The diagnostic performances of the clinical, handcrafted radiomics, DLR, and final Combined models were evaluated using ROC analysis. As intended by the rigorous model selection design to prevent data leakage, the optimal architecture was confirmed based strictly on the internal validation cohort.

**Figure 4:**
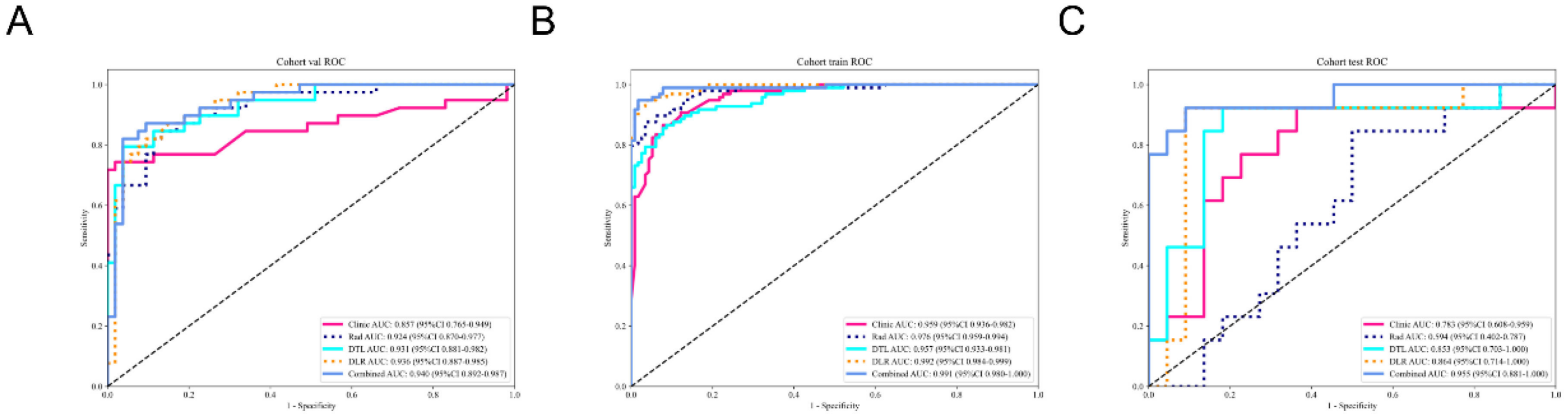
Different signatures of receiver operating characteristic (ROC) curves on different cohorts. The Area Under the Curve (AUC) for the Clinical, Handcrafted Radiomics (Rad), Deep Transfer Learning (DTL), Deep Learning Radiomics (DLR), and Combined models across the **(A)** Training cohort, **(B)** Internal Validation cohort, and **(C)** External Testing cohort.

The integration of clinical predictors with the DLR signature yielded the strongest predictive performance. The Combined Model achieved an AUC of 0.991 (95% CI: 0.980–1.000) in the training cohort, 0.940 (95% CI: 0.892–0.987) in the internal validation cohort, and 0.955 (95% CI: 0.882–1.000) in the external testing cohort. These high AUC metrics demonstrate that the Combined Model can highly accurately distinguish endometrial cancer from simple endometrial hyperplasia and endometrial polyps (**Table 4**; **Figure 4**).

**Table 4:** Diagnostic performance metrics of the clinical, radiomics, deep transfer learning (DTL), deep learning radiomics (DLR), and combined models across all cohorts.

Signature	Accuracy	AUC	95% CI	Sensitivity	Specificity	PPV	NPV	Cohort
Clinic	0.892	0.959	0.9359 - 0.9821	0.856	0.922	0.902	0.883	train
Rad	0.92	0.976	0.9593 - 0.9936	0.866	0.965	0.955	0.895	train
DTL	0.887	0.957	0.9331 - 0.9805	0.856	0.913	0.892	0.882	train
DLR	0.953	0.992	0.9841 - 0.9990	0.938	0.965	0.958	0.949	train
Combined	0.962	0.991	0.9800 - 1.0000	0.938	0.983	0.978	0.95	train
Clinic	0.87	0.857	0.7646 - 0.9494	0.718	0.981	0.966	0.825	val
Rad	0.848	0.924	0.8704 - 0.9767	0.821	0.868	0.821	0.868	val
DTL	0.88	0.931	0.8810 - 0.9816	0.769	0.962	0.937	0.85	val
DLR	0.859	0.936	0.8867 - 0.9851	0.795	0.906	0.861	0.857	val
Combined	0.891	0.94	0.8920 - 0.9871	0.795	0.962	0.939	0.864	val
Clinic	0.714	0.783	0.6079 - 0.9585	0.846	0.636	0.579	0.875	test
Rad	0.6	0.594	0.4020 - 0.7868	0.769	0.5	0.476	0.786	test
DTL	0.829	0.853	0.7030 - 1.0000	0.846	0.818	0.733	0.9	test
DLR	0.886	0.864	0.7137 - 1.0000	0.846	0.909	0.846	0.909	test
Combined	0.886	0.955	0.8815 - 1.0000	0.846	0.909	0.846	0.909	test

Rad, handcrafted radiomics signature; DTL, deep transfer learning signature (ResNet152 backbone); DLR, deep learning radiomics signature; AUC, area under the curve; Acc, accuracy; Sens, sensitivity; Spec, specificity; PPV, positive predictive value; NPV, negative predictive value.

### 3.6 Clinical application value of each model

**Figure 5:**
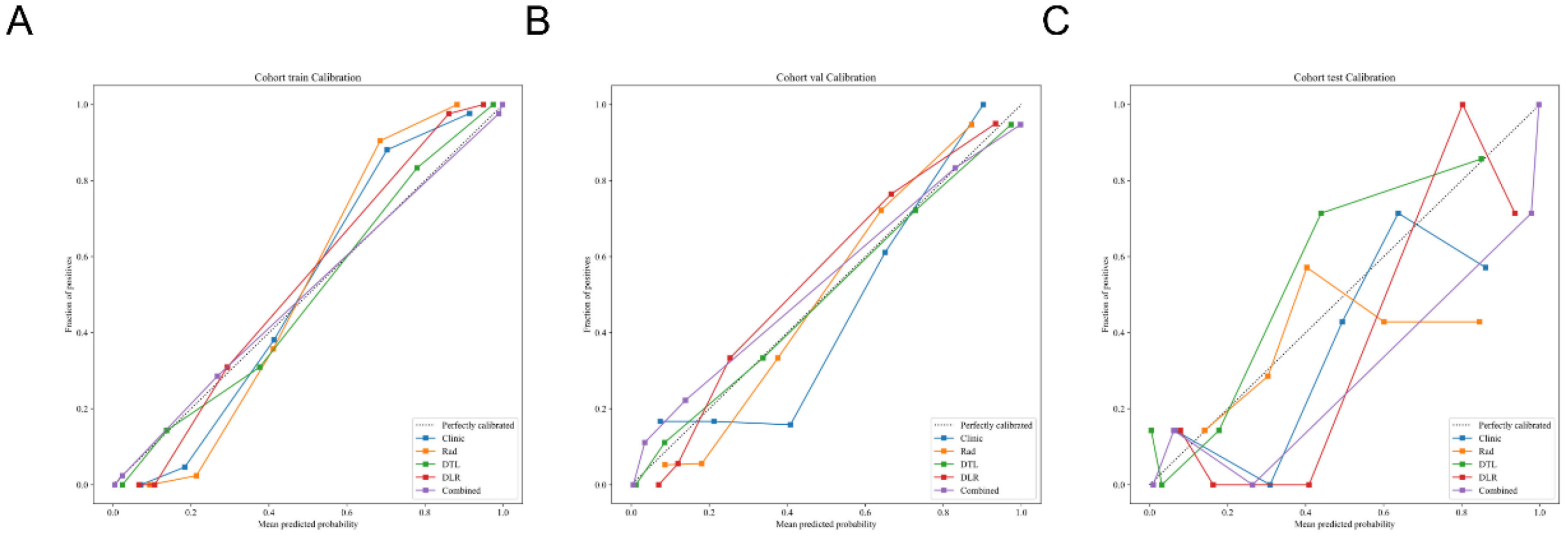
Calibration curves of different signatures for the different cohorts. **(A)** Training cohort, **(B)** Internal Validation cohort, and **(C)** External Testing cohort.

Calibration curves: The calibration of the models was evaluated using the Hosmer-Lemeshow test. As shown in **Figure 5**, the Combined Model demonstrated excellent agreement between the predicted probabilities and actual observations across all cohorts, with no significant deviation globally.

**Figure 6:**
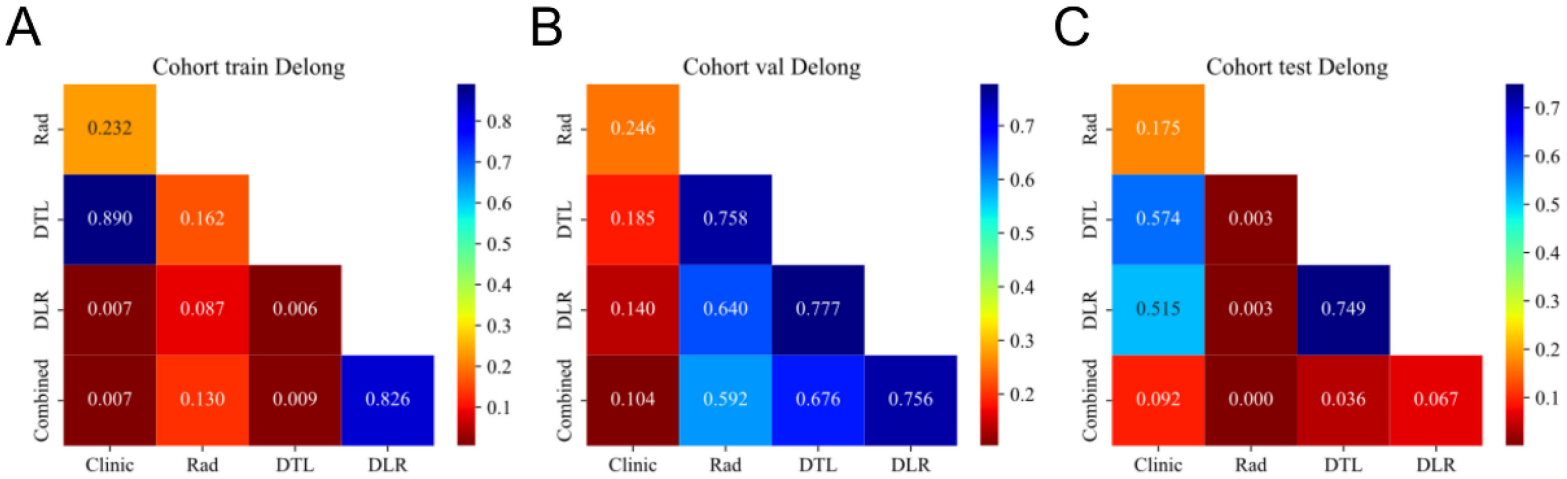
Heatmap visualization of the DeLong test results for different signatures. **(A)** Training cohort, **(B)** Internal Validation cohort, and **(C)** External Testing cohort.

DeLong test: We employed the DeLong test to statistically compare the AUC values. As illustrated in **Figure 6**, within the independent testing cohort, the Combined Model demonstrated a statistically significant improvement in performance compared to both the standalone handcrafted Radiomics Model and the Clinical Model. These results underscore that fusing multimodal imaging data with comprehensive clinical features significantly enhances predictive robustness.

**Figure 7:**
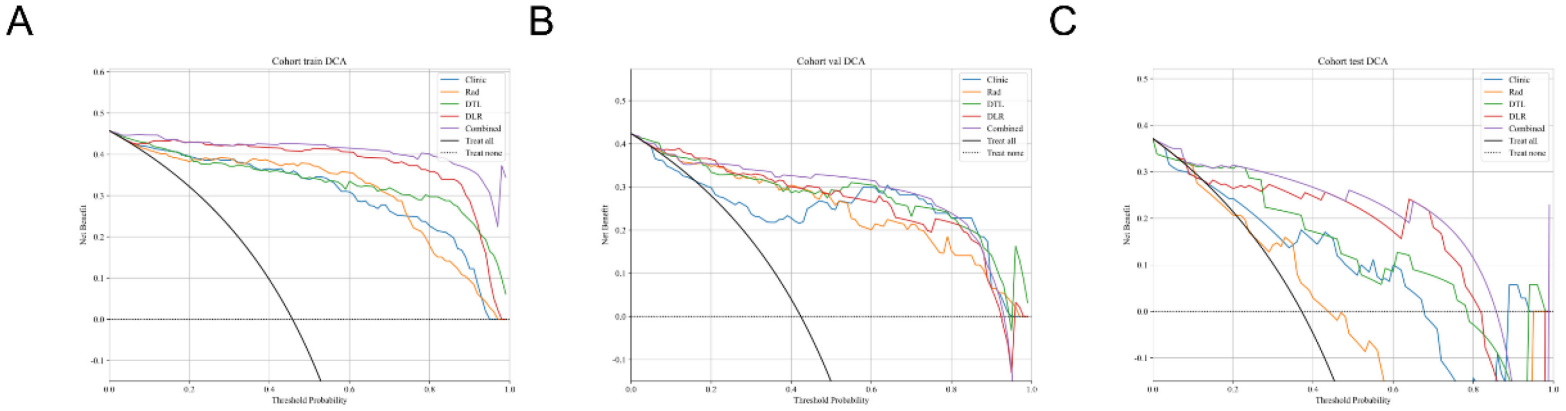
Decision curve analysis **(DCA)** of the different signatures for the different cohorts. **(A)** Training cohort, **(B)** Internal Validation cohort, and **(C)** External Testing cohort.

**Figure 8:**
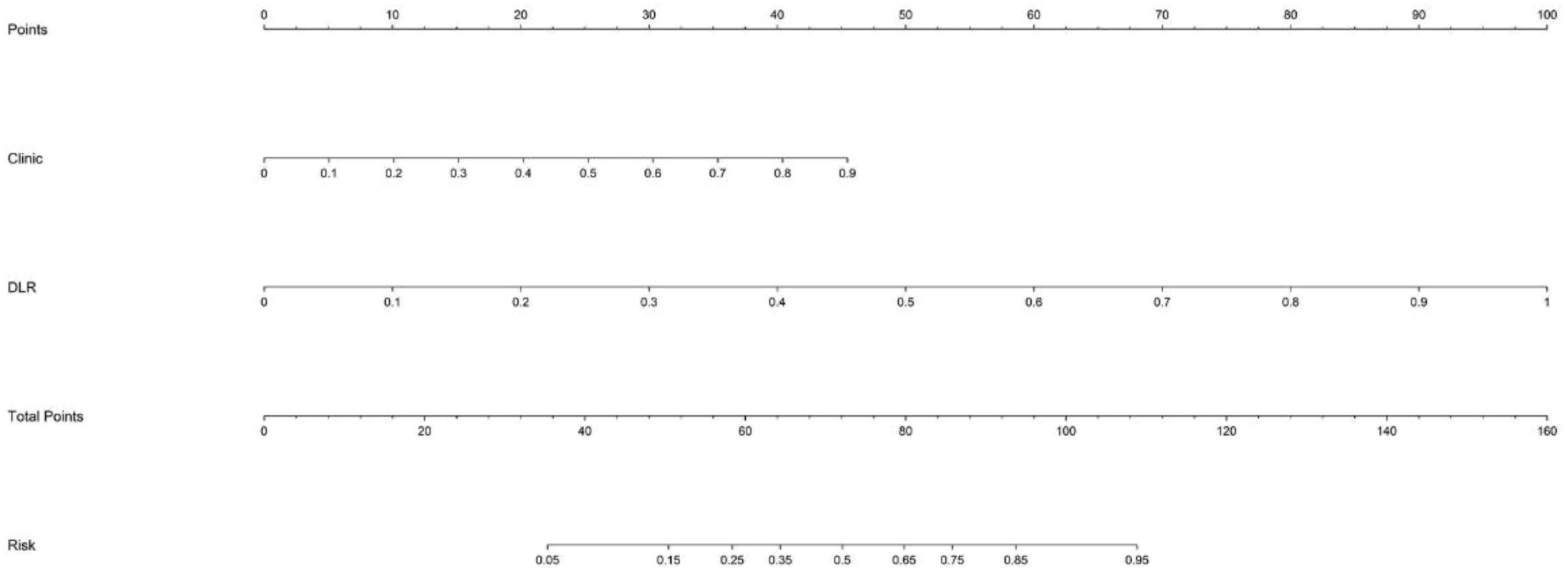
The developed nomogram for predicting endometrial cancer risk.

Decision curve analysis (DCA) and Nomogram: **Figure 7** presents the decision curves for the cohorts. The Combined Model exhibited the highest net benefit across the majority of threshold probabilities compared to individual feature models, confirming its superior clinical utility. Finally, to facilitate clinical application, a multimodal nomogram integrating the DLR signature and the significant clinical predictors was constructed (**Figure 8**), providing an intuitive and quantitative tool for individualized risk estimation of endometrial lesions.

## 4 Discussion

This study established a benign-malignant differential diagnosis model for 340 patients with endometrial lesions. The model was based on a radiomics machine learning approach using transvaginal ultrasound images. The research first selected original image features and constructed four models: a clinical model, a handcrafted radiomics model, a deep learning model, and a combined model integrating handcrafted radiomics and deep learning. The predictive performance of the models was analyzed using ROC, calibration, and decision curves, and the one with the highest AUC was selected as optimal. Additionally, the study performed univariate and stepwise multivariate regression analyses on clinical features to construct a Combined Signature. The Combined Model achieved AUCs of 0.991 (95% CI: 0.980–1.000) on the training cohort, 0.940 (95% CI: 0.892–0.987) on the internal validation cohort, and 0.955 (95% CI: 0.882–1.000) on the external testing cohort, demonstrating excellent predictive capability for differentiating endometrial cancer from simple endometrial hyperplasia with endometrial polyps. These results indicate that integrating complementary information from clinical variables, handcrafted radiomics, and deep transfer learning features can substantially improve diagnostic performance compared with relying on a single type of feature alone.

Endometrial cancer (EC) is one of the most common malignant tumors in the female reproductive system, and its early diagnosis is crucial for improving patient survival rates (16). Endometrial cancer is typically classified into two types: Type I, which is often associated with estrogen excess and has a better prognosis, and Type II, which is linked to higher tumor stages and poorer prognosis (17). According to the International Federation of Gynecology and Obstetrics (FIGO) staging system, endometrial cancer staging is based on the depth of myometrial invasion and lymph node involvement, which is crucial for treatment planning and prognosis assessment (18). Currently, imaging examinations such as ultrasound, CT, and MRI play important roles in the diagnosis of endometrial cancer (19). Ultrasound, as the most commonly used initial screening tool, can not only assess endometrial thickness and morphological changes (20), but also effectively improve the early detection rate and sensitivity of endometrial cancer when combine with clinical symptoms (21, 22). MRI provides more precise anatomical information, helping physicians assess tumor invasion depth and lymph node metastasis (23, 24). Clinically, the diagnosis of endometrial cancer primarily relies on pathological examination and imaging techniques, but these approaches still have limitations in accuracy and timeliness for early diagnosis. Although numerous studies have explored the clinical and imaging features of endometrial cancer, effective tools for early differentiation from other pathological conditions, such as endometrial hyperplasia and polyps, remain lacking, which poses significant challenges for clinicians during screening and diagnosis (25). Therefore, developing an effective diagnostic tool to improve the early identification rate and treatment efficacy of endometrial cancer is particularly necessary.

In recent years, radiomics, as an emerging technology, has gradually demonstrated its unique advantages in the early diagnosis of tumors. Radiomics provides richer information than traditional imaging by extracting a large number of quantitative features from medical images. This allows physicians to more accurately assess tumor characteristics and prognosis (11, 26). Studies have shown that combining radiomics features with clinical data can significantly improve tumor identification rates and staging accuracy. For example, models based on radiomics features from multiparametric MRI can predict tumor invasion depth, lymph node metastasis, preoperative stage, and risk stratification with AUC values above 0.85, showing high accuracy (27, 28). Our findings are consistent with these previous reports. However, compared with MRI-based studies, our results are noteworthy because our model was developed using TVUS, which is more widely available, less expensive, and more practical for first-line gynecologic evaluation. Despite using a more accessible imaging modality, our Combined Model achieved an AUC of 0.955 in the external testing cohort, suggesting that TVUS-based multimodal modeling may provide diagnostic accuracy comparable to, or even better than, some MRI-based approaches reported in the literature.

With advances in artificial intelligence, researchers have gradually applied deep learning-based image analysis methods to cancer diagnosis. For example, researchers have developed unique deep learning ensemble models based on clinical decision support systems that can accurately and rapidly diagnose breast cancer (29). Furthermore, physicians can identify risk factors associated with tumor development by analyzing patients’ clinical data. This enables personalized treatment plans. For instance, studies show that clinical features such as age, tumor grade, and lymph node metastasis status are closely related to patient prognosis (30, 31). These features not only help assess patient survival rates but also guide clinical decision-making and optimize treatment strategies. In tumor prediction models, the integration of clinical features can significantly improve prediction accuracy (32, 33). By integrating clinical and radiomics features, researchers have built precise models that effectively predict lymph node metastasis in endometrial cancer, showing strong clinical potential (34, 35). Inspired by these findings, we also recognize that integrating clinical features is a key factor contributing to the success of our model. In our study, the inclusion of clinical information, such as age and menopausal status, improved the diagnostic capability of the radiomics framework and likely contributed to the superior performance of the Combined Model. Our analysis confirmed that combining clinical data, such as patient age and menopausal status, with radiomics significantly improved prediction accuracy. Our combined model achieved higher diagnostic accuracy and better overall performance by simulating the clinical reasoning process of experienced radiologists, who always consider patient history alongside imaging findings. These outperformed models based solely on imaging features.

Machine learning algorithms can significantly improve the speed and accuracy of image analysis through automated feature extraction and pattern recognition (36). For example, deep learning models achieve higher accuracy than human experts in tumor detection and classification, particularly when analyzing complex imaging data (37). In radiomics feature extraction, machine learning techniques can extract a large number of quantitative features from medical images, which can be used to predict the biological characteristics and clinical outcomes of diseases (38). Integrating radiomics features with clinical data enables researchers to build more precise predictive models. Models that combine deep learning with radiomics features demonstrate strong performance in predicting myometrial invasion, clinical risk category, histological type, and lymphovascular space invasion in endometrial cancer, highlighting their clinical potential (27). This study collected a large number of transvaginal ultrasound images of the endometrium and constructed a Combined Model by integrating deep learning algorithms, clinical features, and handcrafted radiomics methods. Our results suggest that deep transfer learning and handcrafted radiomics provided complementary rather than redundant information. Our model combines deep learning (DL) features with interpretable handcrafted features, capturing the texture heterogeneity and subtle morphological differences of endometrial lesions, thereby improving diagnostic sensitivity. This model significantly improves the accuracy of early diagnosis for endometrial cancer.

Model evaluation is a key aspect of machine learning in medical image analysis. Researchers assess the generalization ability and clinical applicability of models by analyzing AUC values on training, validation, and external testing cohorts; the results demonstrate that these models have good stability and reliability (39, 40). Furthermore, calibration curve analysis provides an important basis for the clinical application of the model, ensuring that the model’s predictions align with actual observations. Decision curve analysis provides an intuitive comparison of the clinical utility of different models (41) and allows researchers to assess the value of combined models versus single models in actual clinical decision-making. For example, combined models show higher net benefit in decision curve analysis, indicating their advantage over single models in clinical application (42, 43). We evaluated the practical clinical value of the model and confirmed its relevance through decision curve analysis (DCA). The results show that, within a range of threshold probabilities, using the combined model to guide biopsy decisions offers a higher net benefit than the “treat all” or “treat none” strategies. In clinical practice, our combined model enables clinicians to accurately identify high-risk patients requiring hysteroscopy and biopsy, while reducing unnecessary invasive procedures for those with benign polyps or simple hyperplasia. Therefore, in addition to its high discriminatory performance, the Combined Model may also serve as a useful noninvasive triage tool in routine clinical practice. Although these results are encouraging, it is important to recognize potential failure modes in TVUS analysis that may affect the model’s performance. For example, false positives may occur in cases of atypical endometrial hyperplasia or large polyps with vascular proliferation, as these conditions produce radiomic features similar to those observed in cancer. Conversely, false negatives may arise in early-stage localized cancer where tissue structures remain relatively intact; in such cases, the model might overlook these subtle radiomic features. However, unlike CT or MRI, TVUS is inherently operator-dependent, and variations in probe pressure, scanning angle, or machine settings can introduce noise into radiomic features. Although our deep learning backbone network helps extract robust features, standardized imaging acquisition protocols are essential for the widespread clinical application of this tool.

This study aimed to construct a predictive model based on radiomics features and deep learning to improve the early diagnosis of endometrial cancer. In this study, we first collected extensive patient clinical and imaging data, extracted radiomics features from transvaginal ultrasound images, and then used deep learning algorithms to build the predictive model. We evaluated the model’s performance using the training cohort, internal validation cohort, and external testing cohort. The model achieved AUC values of 0.991, 0.94, and 0.955 on the three datasets, demonstrating strong predictive performance. The results of this study not only validate the effectiveness of radiomics features combined with deep learning in predicting endometrial cancer but also provide specific new directions for future research, namely combining multimodal imaging data and clinical features to further enhance the accuracy and generalization ability of predictive models. Future research should explore the combination of radiomics features with other clinical features to optimize model performance and validate the model’s applicability in larger-scale clinical samples. It is worth noting that recent studies have explored other advanced techniques. For example, peritumoral-based models can capture valuable microenvironmental features. Additionally, Multiple Instance Learning (MIL) approaches have shown promise in weak supervision tasks (44, 45). In comparison, our study demonstrates that explicitly fusing Deep Transfer Learning with interpretative clinical and radiomics data, as done in our Combined Model, yields highly competitive and robust diagnostic performance (AUC = 0.955). This approach potentially offers superior stability for clinical applications compared to single-modality methods.

This study has several limitations. First, as a retrospective study, selection bias may affect model accuracy. Second, the sample size is relatively limited, especially in the external testing cohort. In scenarios with small samples and high feature-to-sample ratios, there is a risk of overfitting. Therefore, larger-scale, multicenter studies are needed to validate the model’s generalizability. Third, inter-reader variability and manual ROI delineation can introduce human error during image analysis. Moreover, future research could integrate magnetic resonance imaging (MRI) into machine learning comparisons to develop improved models. Finally, ultrasound image quality depends heavily on operator skill and equipment performance, which may impact the stability and reproducibility of radiomics analysis. Thus, establishing standardized protocols for image acquisition and analysis is essential to minimize technical variability.

## 5 Conclusion

In conclusion, this study successfully built a predictive model for endometrial cancer based on radiomics features and deep learning. The model demonstrated potential in early diagnosis and can serve as a preliminary clinical decision support tool. Due to the small sample size, readers should interpret the results with caution, and further validation is needed before wide clinical application. Furthermore, we anticipate that future research will improve the model’s prediction accuracy and clinical utility. This improvement will more effectively support the early diagnosis and treatment of patients with endometrial cancer.

## Data Availability

The raw data supporting the conclusions of this article will be made available by the authors, without undue reservation.

## Supplementary material

Supplementary Figure 1 Feature selection using the least absolute shrinkage and selection operator (LASSO) logistic regression model. **(A, B)** Selection of the optimal tuning parameter (λ) in the LASSO model using 10-fold cross-validation via minimum criteria. The y-axis indicates the Mean Squared Error (MSE). The vertical dotted lines represent the optimal λ values of 0.0256 for Deep Learning Radiomics (DLR) features **(A)** and 0.0518 for Handcrafted Radiomics features **(B)**, where the optimal number of features were selected. **(C, D)** LASSO coefficient profiles of the DLR features ^©^ and Handcrafted Radiomics features **(D)**. Each colored line represents the coefficient of an individual feature. As λ increases (x-axis), coefficients are compressed towards zero. The vertical dotted lines indicate the optimal λ values chosen by cross-validation, defining the final subset of features with non-zero coefficients.

Supplementary Table 1 Detailed configurations of the Deep Learning search space and Machine Learning hyperparameters.

Supplementary Table 2 Detailed list of selected features and their corresponding coefficients derived from the LASSO logistic regression model. Data were obtained using the least absolute shrinkage and selection operator (LASSO) logistic regression model with 10-fold cross-validation. Section **(A)** lists the handcrafted radiomics features, and Section **(B)** lists the combined features (deep learning features + radiomics features) for the DLR signature. The prediction score is calculated using the formula: Score=Intercept+∑(Feature×Coefficient). Features are named according to the PyRadiomics standard (Filter_Class_Feature). DLR, deep learning radiomics; GLCM, gray-level co-occurrence matrix; GLSZM, gray-level size zone matrix; NGTDM, neighboring gray tone difference matrix; GLRLM, gray-level run length matrix; LBP, local binary pattern.

Supplementary Table 3 Results of 17 machine models in 10 deep learning series.
