# Supplementary material for: A combined model based on clinical, radiomics, and deep transfer learning features for differentiating endometrial hyperplasia with polyps from endometrial cancer
Source: Front Oncol. 2026 Jul 28;16:1818266. doi: 10.3389/fonc.2026.1818266 (PMC13457211; doi:10.3389/fonc.2026.1818266)
Supplement: Supplementary file 1 [file Image1.pdf]

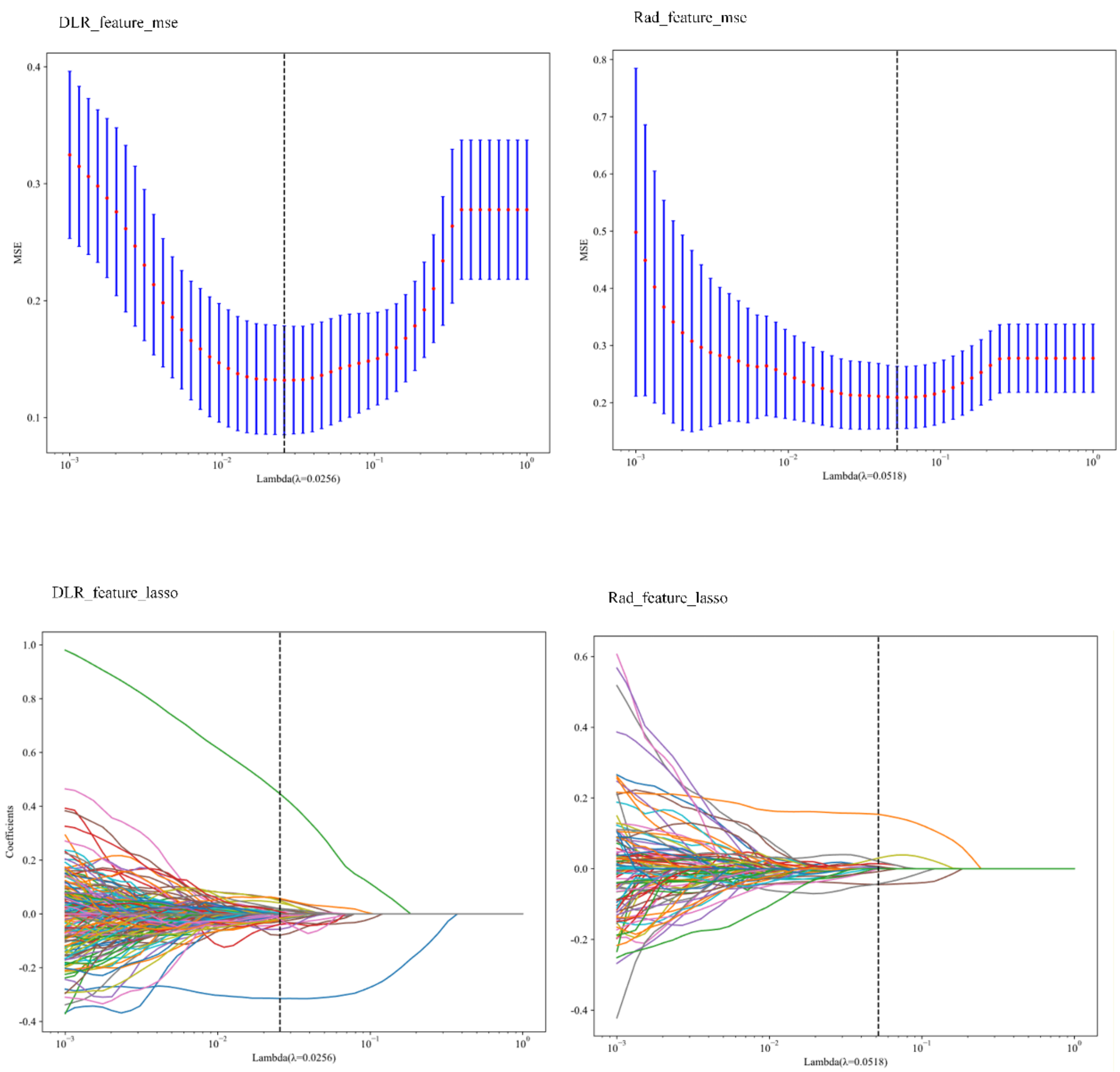

Supplementary Figure S1. Feature selection using the Least Absolute Shrinkage and Selection Operator (LASSO) logistic regression model.

(A, B) Selection of the optimal tuning parameter (λ) in the LASSO model using 10-fold cross-validation via minimum criteria. The y-axis indicates the Mean Squared Error (MSE). The vertical dotted lines represent the optimal λ values of 0.0256 for Deep Learning Radiomics (DLR) features (A) and 0.0518 for Handcrafted Radiomics features (B), where the optimal number of features were selected.

(C, D) LASSO coefficient profiles of the DLR features © and Handcrafted Radiomics features (D). Each colored line represents the coefficient of an individual feature. As λ increases (x-axis), coefficients are compressed towards zero. The vertical dotted lines indicate the optimal λ values chosen by cross-validation, defining the final subset of features with non-zero coefficients.
