# Supplementary material for: A combined model based on clinical, radiomics, and deep transfer learning features for differentiating endometrial hyperplasia with polyps from endometrial cancer
Source: Front Oncol. 2026 Jul 28;16:1818266. doi: 10.3389/fonc.2026.1818266 (PMC13457211; doi:10.3389/fonc.2026.1818266)
Supplement: Supplementary file 2 [file Table1.pdf]

Supplementary Table S1. Detailed configurations of the Deep Learning search space and Machine Learning hyperparameters.

Models / Parameters	Configurations / Architectures
**Deep Learning Search Space (17 Architectures)**	
ResNet family	ResNet18, ResNet34, ResNet50, ResNet101, ResNet152 (Selected)
DenseNet family	DenseNet121, DenseNet161, DenseNet169, DenseNet201
Classic architectures	AlexNet, VGG19, Inception_v3
Lightweight models	MobileNet_v2, MnasNet0_5, ShuffleNet_v2_x1_0, SqueezeNet1_1
Transformer-based	Vision Transformer (ViT): patch_size=64, dim=1024, depth=6, heads=16
**Deep Learning Training Hyperparameters**	
Optimizer	Stochastic Gradient Descent (SGD)
Initial learning rate	0.01
Batch size	32
Epochs	100
Regularization	Dropout rate = 0.5, Batch Normalization
Weight initialization	ImageNet pre-trained weights
**Machine Learning Classifiers (8 Algorithms)**	
Logistic Regression (LR)	penalty = none, max_iter = 100
Support Vector Machine (SVM)	probability = True, max_iter = 100
K-Nearest Neighbors (KNN)	n_neighbors = 5, metric = minkowski
Random Forest (RF)	n_estimators = 50, max_depth = 5, min_samples_split = 4
Extra Trees	n_estimators = 20, max_depth = 5, min_samples_split = 2
XGBoost	n_estimators = 100, objective = binary: logistic, max_depth = 3, min_child_weight = 0.2
LightGBM	n_estimators = 20, max_depth = 4, min_child_weight = 0.5
Multi-Layer Perceptron (MLP)	hidden_layer_sizes = (100,), activation = relu, solver = adam, max_iter = 200
