# Supplementary material for: A combined model based on clinical, radiomics, and deep transfer learning features for differentiating endometrial hyperplasia with polyps from endometrial cancer
Source: Front Oncol. 2026 Jul 28;16:1818266. doi: 10.3389/fonc.2026.1818266 (PMC13457211; doi:10.3389/fonc.2026.1818266)
Supplement: Supplementary file 3 [file Table2.pdf]

Supplementary Table S2. Detailed list of selected features and their corresponding coefficients derived from the LASSO logistic regression model.

Feature Name	Coefficient
**A. Radiomics Signature**	
Intercept	0.441315
original_shape_Elongation	0.154016
original_glcm_Idn	0.029945
wavelet_LLL_glcm_Idn	0.020832
wavelet_LHH_ngtdm_Contrast	0.014238
gradient_glcm_Idn	0.006539
exponential_ngtdm_Busyness	0.003814
squareroot_glcm_Imc1	0.000893
wavelet_LHL_glcm_Correlation	-0.008276
original_glcm_DifferenceEntropy	-0.043121
logarithm_glszm_SmallAreaEmphasis	-0.044812
**B. DLR Signature**	
Intercept	0.441315
DL_1	0.049465
DL_5	0.036385
DL_3	0.027622
wavelet_HLL_ngtdm_Coarseness	0.026721
squareroot_glcm_Idn	0.026311
wavelet_HLL_glcm_InverseVariance	0.019342
wavelet_HHH_ngtdm_Contrast	0.0175
original_shape_Elongation	0.007306
gradient_glcm_Correlation	0.006276

wavelet_HHH_glszm_SizeZoneNonUniformity	0.005636
wavelet_LLL_ngtdm_Coarseness	0.00491
squareroot_glcm_Id	0.003672
wavelet_HLL_glcm_ClusterShade	0.003502
DL_2	0.000559
wavelet_HLL_glcm_Imc2	-0.000455
wavelet_LLH_glrlm_LowGrayLevelRunEmphasis	-0.001823
wavelet_HHL_glcm_Imc2	-0.00414
exponential_glcm_InverseVariance	-0.004435
lbp_3D_k_ngtdm_Strength	-0.004681
wavelet_LHL_firstorder_Median	-0.007489
wavelet_HHH_glszm_LowGrayLevelZoneEmphasis	-0.017243
wavelet_HLH_firstorder_Mean	-0.024952
wavelet_LHH_glszm_ZoneEntropy	-0.02528
DL_0	-0.31895
DL_6	-0.042319

Data were obtained using the least absolute shrinkage and selection operator (LASSO) logistic regression model with 10-fold cross-validation. Section **(A)** lists the handcrafted radiomics features, and Section **(B)** lists the combined features (deep learning features + radiomics features) for the DLR signature. The prediction score is calculated using the formula: $Score = Intercept + \sum(Feature \times Coefficient)$.

Note: Features are named according to the PyRadiomics standard (Filter_Class_Feature).

Abbreviations: DLR, deep learning radiomics; GLCM, gray-level co-occurrence matrix; GLSZM, gray-level size zone matrix; NGTDM, neighboring gray tone difference matrix; GLRLM, gray-level run length matrix; LBP, local binary pattern.
