# Supplementary material for: A combined model based on clinical, radiomics, and deep transfer learning features for differentiating endometrial hyperplasia with polyps from endometrial cancer
Source: Front Oncol. 2026 Jul 28;16:1818266. doi: 10.3389/fonc.2026.1818266 (PMC13457211; doi:10.3389/fonc.2026.1818266)
Supplement: Supplementary file 4 [file Table3.pdf]

Supplementary table S3. Results of 17 machine models in 10 deep learning series.

Model Name	Acc	AUC	95% CI	Sensitivity	Specificity	PPV	NPV	Precision	Recall	F1	Threshold	Cohort
densenet121	0.882	0.949	0.9220-0.9766	0.856	0.904	0.883	0.881	0.883	0.856	0.869	0.408	Train
densenet161	0.792	0.854	0.8031-0.9048	0.794	0.791	0.762	0.82	0.762	0.794	0.778	0.433	Train
densenet169	0.873	0.957	0.9348-0.9793	0.979	0.783	0.792	0.978	0.792	0.979	0.876	0.258	Train
densenet201	0.844	0.921	0.8859-0.9562	0.804	0.878	0.848	0.842	0.848	0.804	0.825	0.456	Train
resnet18	0.877	0.946	0.9173-0.9748	0.876	0.878	0.859	0.894	0.859	0.876	0.867	0.489	Train
resnet34	0.887	0.944	0.9139-0.9733	0.814	0.948	0.929	0.858	0.929	0.814	0.868	0.659	Train
resnet50	0.802	0.882	0.8378-0.9264	0.742	0.852	0.809	0.797	0.809	0.742	0.774	0.486	Train
resnet101	0.896	0.966	0.9466-0.9854	0.887	0.904	0.887	0.904	0.887	0.887	0.887	0.428	Train
resnet152	0.887	0.957	0.9331-0.9805	0.856	0.913	0.892	0.882	0.892	0.856	0.874	0.499	Train
inception_v3	0.745	0.801	0.7416-0.8611	0.68	0.8	0.742	0.748	0.742	0.68	0.71	0.583	Train
vgg19	0.863	0.883	0.8350-0.9314	0.794	0.922	0.895	0.841	0.895	0.794	0.842	0.502	Train
VIT	0.613	0.627	0.5524-0.7021	0.649	0.583	0.568	0.663	0.568	0.649	0.606	0.432	Train
alexnet	0.774	0.844	0.7924-0.8956	0.794	0.757	0.733	0.813	0.733	0.794	0.762	0.389	Train
squeezenet1_1	0.755	0.839	0.7854-0.8925	0.773	0.739	0.714	0.794	0.714	0.773	0.743	0.423	Train
mobilenet_v2	0.882	0.957	0.9342-0.9796	0.959	0.817	0.816	0.959	0.816	0.959	0.882	0.32	Train
mnasnet0_5	0.566	0.594	0.5174-0.6709	0.732	0.426	0.518	0.653	0.518	0.732	0.607	0.059	Train
shufflenet_v2_x1_0	0.509	0.485	0.4072-0.5631	0.608	0.426	0.472	0.563	0.472	0.608	0.532	0.494	Train
densenet121	0.843	0.893	0.8326-0.9538	0.846	0.84	0.786	0.887	0.786	0.846	0.815	0.488	Test
densenet161	0.858	0.871	0.8020-0.9409	0.769	0.92	0.87	0.852	0.87	0.769	0.816	0.5	Test
densenet169	0.858	0.903	0.8469-0.9585	0.808	0.893	0.84	0.87	0.84	0.808	0.824	0.429	Test

densenet201	0.843	0.893	0.8312-0.9540	0.788	0.88	0.82	0.857	0.82	0.788	0.804	0.588	Test
resnet18	0.835	0.895	0.8359-0.9546	0.827	0.84	0.782	0.875	0.782	0.827	0.804	0.502	Test
resnet34	0.835	0.888	0.8289-0.9470	0.75	0.893	0.83	0.837	0.83	0.75	0.788	0.536	Test
resnet50	0.874	0.894	0.8314-0.9573	0.808	0.92	0.875	0.873	0.875	0.808	0.84	0.565	Test
resnet101	0.858	0.899	0.8423-0.9548	0.808	0.893	0.84	0.87	0.84	0.808	0.824	0.519	Test
resnet152	0.85	0.908	0.8550-0.9619	0.692	0.96	0.923	0.818	0.923	0.692	0.791	0.613	Test
inception_v3	0.819	0.855	0.7856-0.9238	0.769	0.853	0.784	0.842	0.784	0.769	0.777	0.44	Test
vgg19	0.843	0.869	0.8027-0.9358	0.769	0.893	0.833	0.848	0.833	0.769	0.8	0.426	Test
VIT	0.654	0.653	0.5560-0.7501	0.481	0.773	0.595	0.682	0.595	0.481	0.532	0.368	Test
alexnet	0.772	0.839	0.7660-0.9112	0.788	0.76	0.695	0.838	0.695	0.788	0.739	0.299	Test
squeezenet1_1	0.803	0.851	0.7847-0.9181	0.788	0.813	0.745	0.847	0.745	0.788	0.766	0.384	Test
mobilenet_v2	0.843	0.885	0.8239-0.9464	0.692	0.947	0.9	0.816	0.9	0.692	0.783	0.589	Test
mnasnet0_5	0.669	0.713	0.6194-0.8060	0.673	0.667	0.583	0.746	0.583	0.673	0.625	0.401	Test
shufflenet_v2_x1_0	0.402	0.371	0.2697-0.4713	0.981	0	0.405	0	0.405	0.981	0.573	0.485	Test
